# Ag Nanowires-Enhanced Sb_2_Se_3_ Microwires/Se Microtube Heterojunction for High Performance Self-Powered Broadband Photodetectors

**DOI:** 10.3390/nano15241849

**Published:** 2025-12-10

**Authors:** Shubin Zhang, Xiaonan Wang, Juntong Cui, Yanfeng Jiang, Pingping Yu

**Affiliations:** School of Integrated Circuits, Jiangnan University, Wuxi 214122, China; shubinzhang@jiangnan.edu.cn (S.Z.); 6253802002@stu.jiangnan.edu.cn (X.W.); 1038220426@stu.jiangnan.edu.cn (J.C.); jiangyf@jiangnan.edu.cn (Y.J.)

**Keywords:** Sb_2_Se_3_ microwires, Se microtube, Ag nanowires, photoelectric performances, heterojunctions, broadband photodetectors

## Abstract

The implementation of photoelectric conversion in photoelectric integrated systems requires the design of photodetectors (PDs) with quick response times and low power consumption. In this work, the self-powered photodetector was prepared by antimony selenide (Sb_2_Se_3_) microwires (MW)/Se microtube (MT) heterojunction by coating Ag nanowires (NW). The incorporation of Ag-NW involves dual enhancement mechanisms. First, the surface plasmon resonance (SPR) effect amplifies the light absorption across UV–vis–NIR spectra, and the conductive networks facilitate the rapid carrier transport. Second, the type-II band alignment between Sb_2_Se_3_ and Se synergistically separates photogenerated carriers, while the Ag-NW further suppress the recombination through built-in electric field modulation. The optimized device achieves remarkable responsivity of 122 mA W^−1^ at 368 nm under zero bias, with a response/recovery time of 8/10 ms, outperforming most reported Sb_2_Se_3_-based detectors. The heterostructure provides an effective strategy for developing self-powered photodetectors with broadband spectral adaptability. The switching ratio, responsivity, and detectivity of the Sb_2_Se_3_-MW/Se-MT/Ag-NW device increased by 260%, 810%, and 849% at 368 nm over the Sb_2_Se_3_-MW/Se-MT device, respectively. These results show that the addition of Ag-NW effectively improves the photoelectric performance of the Sb_2_Se_3_-MW/Se-MT heterojunction, providing new possibilities for the application of self-powered optoelectronic devices.

## 1. Introduction

Photodetectors (PDs) convert optical signals into electrical signals, acting as an indispensable key component in modern optoelectronic systems [1,2,3,4]. According to the spectral response range, standard PDs can be divided into three categories: ultraviolet (UV) light (200–400 nm), visible light (400–760 nm), and near-infrared light (760–1000 nm) [5]. However, traditional PDs with external power sources exhibit significantly low portability and energy efficiency. In addition, scalable deployment is particularly hampered by high drive voltage, which can reach hundreds of volts for UV detectors. To remove the external power, emerging self-powered architectures, which take advantage of the built-in electric fields, provide revolutionary alternatives. Heterojunction-based solutions, which combine multispectral photon capture with interfacial energy band regulation, have great application potentials [6,7,8]. However, to achieve high responsiveness over a wide spectral range without the need for external bias still remains a major challenge. Innovative material systems like Sb_2_Se_3_ micro–nanostructures are of high value when fabricating self-powered broadband PDs [9,10,11,12,13,14]. Sb_2_Se_3_ exhibits high carrier transfer efficiency, strong light absorption capacity, a suitable band structure, and adjustable physical properties, making it suitable for use in PDs with high responsivity and sensitivity [15].

Among the Sb_2_Se_3_ micro–nanostructures, the one-dimensional crystal structure of Sb_2_Se_3_ can more easily obtain high-quality micro–nanostructures [16]. Highly crystalline Sb_2_Se_3_ nanowires (NW) prepared by a hydrothermal method showed a good response to visible light and a fast response speed of less than 0.3 s [17]. Under 3 V bias and 600 nm light irradiation, the responsiveness and external quantum efficiency is 8.0 A W^−1^ and 1650%, respectively. Liu et al. prepared uniform-sized Sb_2_Se_3_ nanowires by the microwave-assisted method and constructed nanowire thin film PDs. The switching ratio and response time of the device under 10 V bias was over 150 and 0.2/1.2s [18]. To further address the shortcomings of Sb_2_Se_3_ micro–nano PDs, such as low responsivity and the absence of self-driving capability, there are numerous reports on the fabrication of Sb_2_Se_3_-based heterojunction PDs [19]. Chen et al. constructed Sb_2_Se_3_/AgSbSe_2_ PDs by a two-step selenization process. Compared with Sb_2_Se_3_ nanorod PDs, the responsivity of this heterojunction PD is improved by 4.2 times, showing excellent photoelectric performance [20]. Overall, these advancements highlight the potential for Sb_2_Se_3_ micro–nanostructures in high-performance photodetection applications, yet disadvantages such as low responsivity and the demand for external power remain to be addressed.

Se, as a p-type inorganic semiconductor material, has a bandgap of about 1.77 eV and a melting point of 217 °C, exhibiting the advantages of low cost, high crystallization performance, and a high response rate [21,22,23]. The response speed of Se-based micro–nanostructure PDs is in the millisecond level. Se microtube (MT) PDs prepared by Hu et al. exhibit a responsivity of 19 mA W^−1^ at 610 nm, with rise and fall times of 0.32 ms and 23.02 ms, respectively [24]. To enhance the responsiveness and response time of Se PD, a common way is to construct a heterojunction with CsPbBr_3_, graphene, PEDOT, InSe, etc. [25,26,27]. Yu et al. fabricated a novel heterojunction structure based on p-type selenium nanoflowers (Se-f) and p-type polyaniline. This device exhibited excellent photoresponse characteristics, particularly achieving a high responsivity of 72.9 mA·W^−1^, a good detectivity of 1.98 × 10^12^ Jones, and fast response times (rise time of 8.6 μs and fall time of 3.24 ms) under unbiased 610 nm illumination [28].

The introduction of metal plasma into Se-based heterojunctions can significantly improve the key performance parameters such as the responsiveness and response speed of PDs. The Local Surface Plasmon Resonance (LSPR) effect is discovered to be crucial in improving the absorption capacity and the local electric field of the devices to generate more photogenerated electrons and holes and further improving the responsivity and self-powered photovoltaic performance [29,30,31]. The noble metals (e.g., Au, Ag, Al) generally exhibit LSPR activity but are limited in practical use by inherent optical losses due to interband electron transitions and free carrier scattering [32,33,34,35]. In particular, silver exhibits superior visible plasmonic efficiency due to resonant matching between its electron cloud oscillation frequency and visible wavelengths, critical for optimal LSPR excitation. Jing et al. achieved a 470% increase in responsivity of 2.97 × 10^4^ A W^−1^ in MoS_2_-based PDs by integrating Ag nanoparticles [36]. Young et al. reported a silver nanoparticle (NPs)/zinc oxide (ZnO) nanorods PD, which exhibited enhanced photoelectrochemical performance with a photocurrent density of 3.22 × 10^−6^ A, and the photoresponsive on/off ratio demonstrated significant improvement from 560 to 5640, which can be attributed to the LSPR effect induced by the incorporated Ag NPs [37]. Therefore, the unique advantages of plasmonic silver nanostructures in UV–visible spectrum detection (350–700 nm), where LSPR effectively promotes the generation of photogenerated electron hole pairs and hot carriers, improves the collection efficiency of photogenerated carriers and greatly enhances the responsiveness and overall photoelectric performance of selenium-based PDs, thus overcoming its performance bottlenecks.

In this work, Se microtubes were prepared by chemical vapor deposition, and we constructed a self-powered heterojunction PD with Sb_2_Se_3_-MW. Ag-NW were coated on the Sb_2_Se_3_-MW/Se-MT device to optimize the photoelectric performances in the UV region. The proposed Sb_2_Se_3_-MW/Se-MT/Ag-NW device exhibits a peak responsivity of 122 mA W^−1^ and a peak detectivity of 1.69 × 10^11^ Jones at 368 nm illumination.

## 2. Materials and Methods

### 2.1. Preparation of the Se-MT

The Se-MT was prepared by chemical vapor deposition in a horizontal tube furnace. A quartz boat with an appropriate amount of Se powder (A.R. 99%) was placed in the center of the tube furnace. A Si/SiO_2_ sheet as a substrate with the size of 2 × 1 cm was washed with acetone, ethanol, and deionized water and then placed 28 cm downstream to the quartz boat. High-purity nitrogen was injected into the horizontal tube furnace at a flow rate of 500 mL min^−1^ for over 20 min to exhaust the air in the furnace. The temperature of the furnace was raised to 300 °C from room temperature in 1 h and maintained for 5 h at a nitrogen flow rate of 200 mL min^−1^. The Se-MT was obtained on the Si/SiO_2_ substrate upon completion of the deposition process.

### 2.2. Preparation of the Sb_2_Se_3_-MW

The Sb_2_Se_3_-MW was prepared by the hydrothermal method. Antimony acetate (Sb(CH_3_COO)_3_, 0.26 g), sodium selenite (Na_2_SeO_3_, 0.85 g), and hydrazine hydrate (0.15 mL, 80 wt%) were dissolved together in deionized water (25 mL) and stirred thoroughly to form a mixed solution. Then, it was transferred to a 30 mL hydrothermal kettle with a Teflon liner, stirred, and then heated to 120 °C in muffle furnace for 8 h. The obtained Sb_2_Se_3_-MW was washed by deionized water and ethanol for several times.

### 2.3. Preparation of the Sb_2_Se_3_-MW/Se-MT and the Sb_2_Se_3_-MW/Se-MT/Ag-NW Heterojunctions

**Figure 1:**
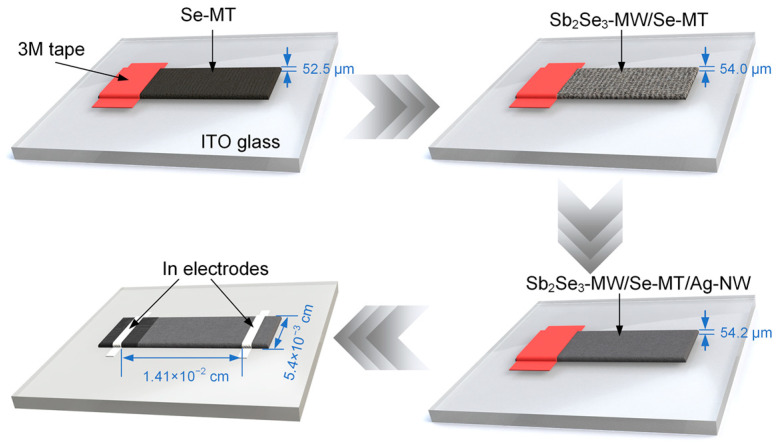
Fabrication flow of the Sb_2_Se_3_-MW/Se-MT/Ag-NW heterojunction.

Figure 1 shows the fabrication of the Sb_2_Se_3_-MW/Se-MT and Sb_2_Se_3_-MW/Se-MT/Ag-NW heterojunctions. The Se-MT on the Si/SiO_2_ substrate was transferred to an Indium–Tin Oxide (ITO) glass, and one end of the Se-MT was covered and fixed by 3M tape. Thus, the rest of the Se-MT was sprayed with the Sb_2_Se_3_-MW solution. After drying for two minutes at 50 °C in a vacuum oven, the Sb_2_Se_3_-MW/Se-MT heterojunction was achieved. Then, with the 3M tape removed and an electrode attached, a PD device was ready for testing. Similarly, to create the Sb_2_Se_3_-MW/Se-MT/Ag-NW heterojunction PD, Ag-NW was further sprayed onto the part where the Sb_2_Se_3_-MW/Se-MT heterojunction formed and was then heated for 5 min at 45 °C. The Ag-NW, with an average diameter of 50 nm and length of 60 μm, were purchased from XFNANO (Nanjing, China) in the form of dispersion (XFJ162, 10 mg mL^−1^). With a typical device area of around 0.0141 cm × 0.0054 cm, 1 μL Ag-NW dispersion was used.

### 2.4. Material Characterization

The morphology of the synthesized Sb_2_Se_3_-MW/Se-MT/Ag-NW nanostructures was analyzed via scanning electron microscopy (SEM), conducted with a JEOL (Tokyo, Japan) JSM-7000F system. Crystalline phase and structural information were obtained through X-ray diffraction (XRD) measurements performed on a Bruker (Berlin, Germany) D8 A25 diffractometer utilizing Cu Kα radiation (λ = 1.5405 Å). The optical absorption characteristics were characterized with a UV–visible spectrophotometer Shimadzu (Kyoto, Japan) UV-2700. The optoelectronic performance and spectral optical responses of the fabricated devices were evaluated utilizing a semiconductor parameter analyzer Keithley (Cleveland, OH, USA) 2636B coupled with a multi-wavelength laser source. The final data were obtained after hundreds of cycles to ensure the accuracy.

## 3. Results and Discussion

**Figure 2:**
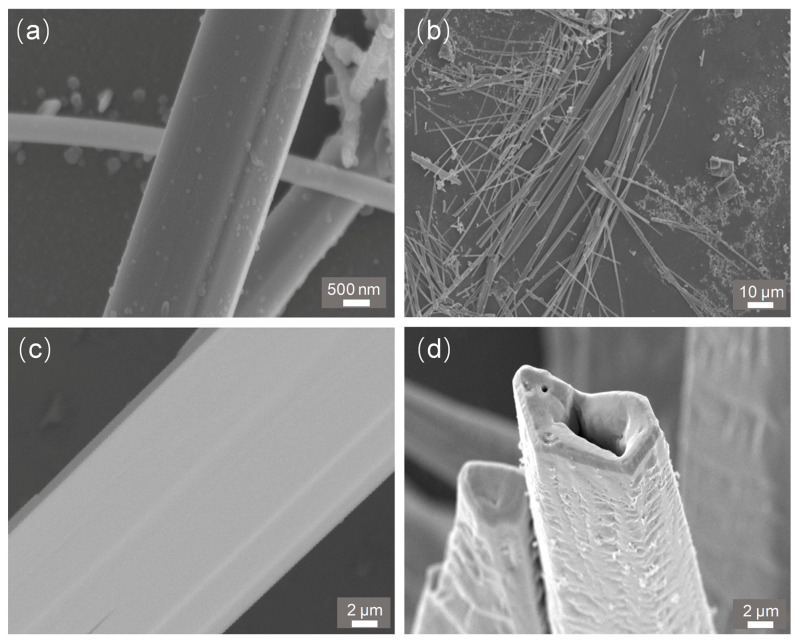
SEM images of the Sb_2_Se_3_-MW with different scale bars: 500 nm (**a**) and 10 μm (**b**) and the surface (**c**) and cross-section (**d**) SEM images of the Se-MT.

Figure 2a shows the SEM image of a single Sb_2_Se_3_ microwire exhibiting a few unreacted particles. Sb_2_Se_3_-MW shows its length in the range of 100 to 200 μm and diameter of about 1 to 4 μm, as displayed in Figure 2b. The Sb_2_Se_3_-MW can be easily transferred to the Se-MT substrate. The Se-MT generated by chemical vapor deposition process is shown in Figure 2c, exhibiting a ultralong tubular form with an external diameter of about 20 μm. The high crystalline Se-MT grew well and exhibits its hexagonal structure with a side length of approximately 5 μm (Figure 2d).

**Figure 3:**
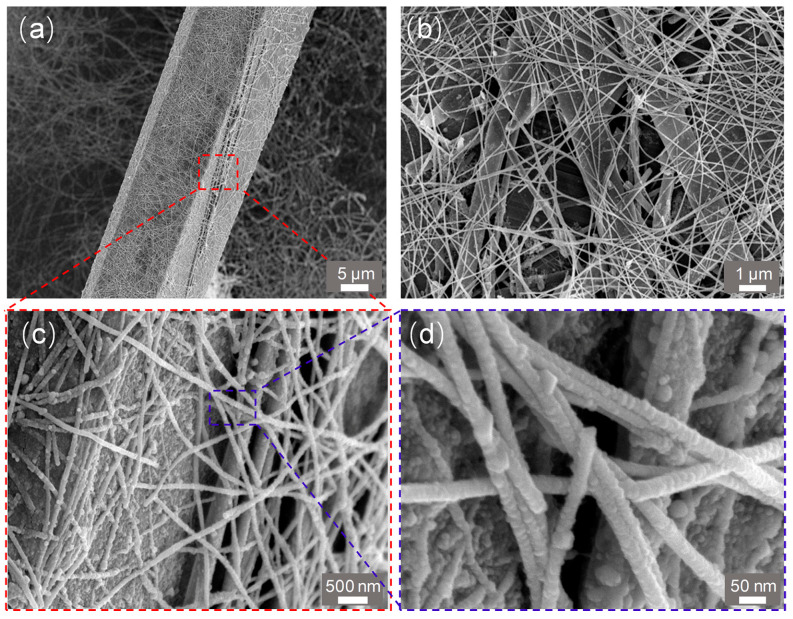
SEM images of the Sb_2_Se_3_-MW/Se-MT/Ag-NW with different scale bars: (**a**) 5 μm, (**b**) 1 μm, (**c**) 500 nm, and (**d**) 50 nm.

After the Ag-NW were sprayed onto the surface of the Sb_2_Se_3_-MW/Se-MT heterojunction, the Sb_2_Se_3_-MW/Se-MT/Ag-NW heterojunction was obtained as shown in Figure 3a. The layer of the Ag-NW evenly wrappers the Sb_2_Se_3_-MW/Se-MT (Figure 3b) like a spider web. Therefore, the heterojunction is exposed and ready to absorb more light. Moreover, through the procedure of fully spraying Ag-NW on Sb_2_Se_3_-MW/Se-MT, good contact among Sb_2_Se_3_-MW, Se-MT, and Ag-NW was guaranteed, as shown in Figure 3c. The Ag-NW exhibit regular fiber morphology with a diameter of about 35–50 nm (Figure 3d).

**Figure 4:**
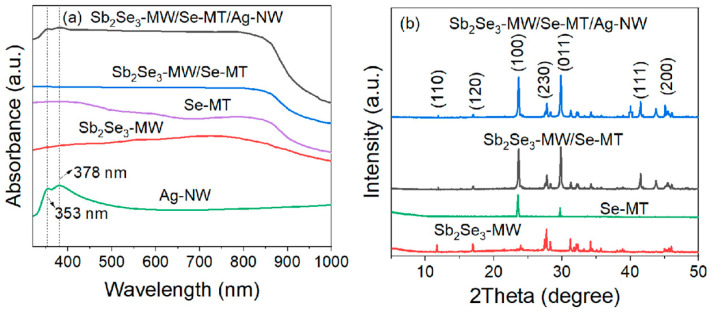
UV–vis absorption spectra of the Ag-NW, Sb_2_Se_3_-MW, Se-MT, Sb_2_Se_3_-MW/Se-MT, and Sb_2_Se_3_-MW/Se-MT/Ag-NW (**a**) and XRD patterns of the Sb_2_Se_3_-MW, Se-MT, Sb_2_Se_3_-MW/Se-MT, and Sb_2_Se_3_-MW/Se-MT/Ag-NW (**b**).

Figure 4a shows the UV–vis absorption spectra of the Ag-NW, Sb_2_Se_3_-MW, Se-MT, Sb_2_Se_3_-MW/Se-MT, and Sb_2_Se_3_-MW/Se-MT/Ag-NW. As can be seen, the Sb_2_Se_3_-MW shows a wide range of absorption from 300 to 1000 nm, culminating at about 750 nm, indicating it is a wide wavelength photoelectric material. Meanwhile, the optical absorption of the Se-MT mainly ranges from 300 to 850 nm, with a significant decrease when the wavelength is bigger than 850 nm. The Sb_2_Se_3_-MW/Se-MT heterojunction exhibits higher light absorption than both the Sb_2_Se_3_-MW and the Se-MT; therefore, it is able to receive more light energy. The Ag-NW absorption curves show two peaks at 353 and 378 nm. After Ag-NW are coated on Sb_2_Se_3_-MW/Se-MT, an absorption enhancement at 353 and 378 nm of the heterojunction appears. This connection exactly corresponds to the transverse and longitudinal plasma resonance peaks of Ag-NW excited by the LSPR effect. The absorption spectra thus show the good performance of the Sb_2_Se_3_-MW/Se-MT/Ag-NW heterojunction. The diffraction peaks of Se-MT, as shown in Figure 4b, correspond to the standard card of No. 65-1876 in the JCPDS library (lattice parameters are a = b = 0.4364 nm, c = 0.4959 nm), which proves the high crystalline of Se-MT. From the XRD spectrum of Sb_2_Se_3_-MW/Se-MT, the diffraction peaks corresponding to the (100) and (011) crystal planes of Se-MT, as well as those corresponding to the (110), (120), (230), (221), (240), (141), (111), (200), and (530) crystal planes of Sb_2_Se_3_-MW, are observed. Compared with Sb_2_Se_3_-MW/Se-MT, the XRD spectrum of Sb_2_Se_3_-MW/Se-MT/Ag-NW showed diffraction peaks belonging to the (111) and (200) crystal planes of Ag-NW, which is consistent with the results of No. 04-0783 in the JCPDS standard card library [38]. This clearly shows that the Sb_2_Se_3_-MW/Se-MT/Ag-NW device was successfully prepared.

**Figure 5:**
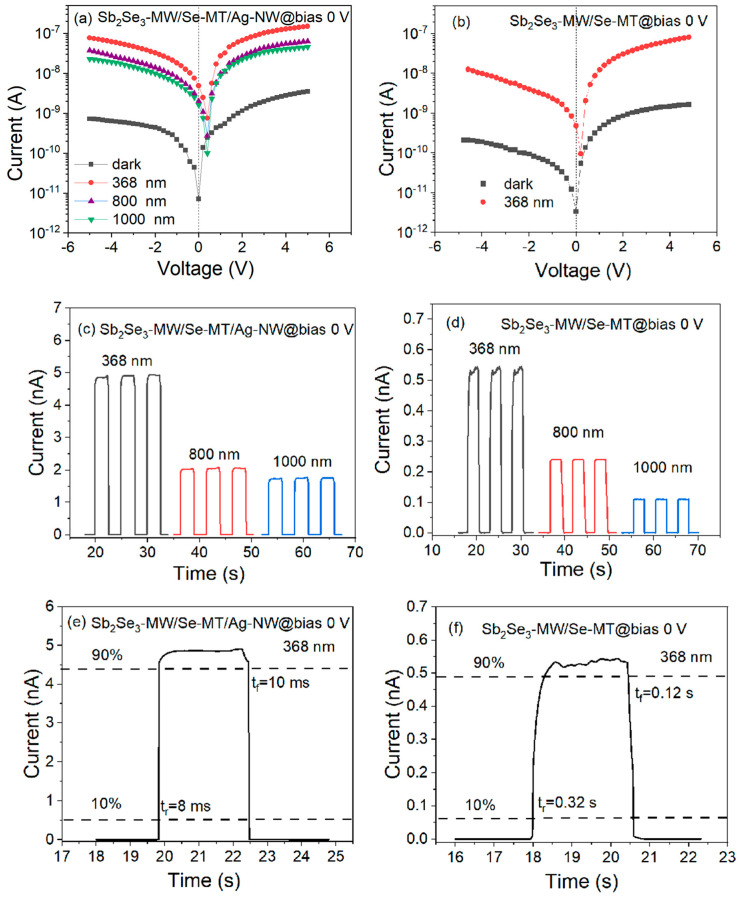
I-V characteristic curves under different illumination conditions of the Sb_2_Se_3_-MW/Se-MT/Ag-NW (**a**) and the Sb_2_Se_3_-MW/Se-MT (**b**). Multi-cycle I-t curves under different illumination conditions of the Sb_2_Se_3_-MW/Se-MT/Ag-NW (**c**) and the Sb_2_Se_3_-MW/Se-MT (**d**). Single-cycle I-t curves under 368 nm illumination of the Sb_2_Se_3_-MW/Se-MT/Ag-NW (**e**) and the Sb_2_Se_3_-MW/Se-MT (**f**).

To further confirm that the enhancement of the photoelectric performance was induced by the LSPR effect, the Sb_2_Se_3_-MW/Se-MT and the Sb_2_Se_3_-MW/Se-MT/Se-MT/Ag-NW heterojunctions were both attached to in electrode to form ohmic contact for the current–voltage (I-V) test. The I-V curves of the Sb_2_Se_3_-MW/Se-MT/Se-MT/Ag-NW under darkness and illumination at 368, 800, and 1000 nm are shown in Figure 5a. The current in darkness is approximately 7.78 × 10^−12^ A and remains the lowest. Under illumination, the open-circuit voltage (V_oc_) indicated by the voltage offset equals about 0.18 V, which resulted from a built-in electric field. Thus, the built-in electric field enables a photoresponse without the requirement for external drives, demonstrating the self-powering capability. The light current (I_light_) in the ultraviolet region (368 nm) is increased by about two orders of magnitude from the dark current and appears to be the highest among all wavelengths. It drops by 50 nA at 800 nm under 5 V bias and then shows a slight decrease when the wavelength reaches 1000 nm. Non-ideally, the I-V curves in logarithmic coordinates at forward and reverse bias appear to be asymmetric. This may mainly result from the low barrier height of the ohmic contact and the different degrees of contact of the In electrode. Nevertheless, with a value of about 2.0, the rectification ratio is negligibly small. Overall, Figure 5a indicates a good broadband photoelectronic performance of the Sb_2_Se_3_-MW/Se-MT/Se-MT/Ag-NW heterojunction. Figure 5b shows the I-V characteristic curves of the Sb_2_Se_3_-MW/Se-MT device under no illumination and 368 nm light sources. By comparison, it can be found that under a 368 nm light source, the I_light_ of the Sb_2_Se_3_-MW/Se-MT/Ag-NW device is much larger than that of the Sb_2_Se_3_-MW/Se-MT device. The introduction of the Ag-NW into the Sb_2_Se_3_-MW/Se-MT heterojunction can greatly enhance the photoresponse of the device.

The current–time (I-t) curves of the two devices are shown in Figure 5c,d, respectively. As can be seen from Figure 5c, the I_light_ of the Sb_2_Se_3_-MW/Se-MT/Ag-NW device under 0 V bias and 368, 800, and 1000 nm light sources is 4.81, 2.09, and 1.72 nA, respectively. Divided by the dark current of 7.78 pA, the corresponding on/off ratios are calculated as 618, 269, and 221, respectively. In Figure 5d, the average I_light_ of the Sb_2_Se_3_-MW/Se-MT device at 0 V bias and 368 nm is about 0.533 nA, and the dark current of it is 3.12 pA (Figure 5b), leading to an on/off ratio of 171. Comparing the on/off ratios at 368 nm, the performance of the Sb_2_Se_3_-MW/Se-MT/Ag-NW device is significantly (260%) higher than that of the Sb_2_Se_3_-MW/Se-MT device. This can be explained by the stronger built-in electric field induced by Ag-NW that separates the electron–hole pairs more efficiently, thus considerably increasing the photocurrent. I-t curves of the devices biased at −4 V are presented as Supporting Information (Figure S1), and similar results are obtained.

Interestingly, the addition of Ag-NW can effectively improve the optoelectronic performances of Sb_2_Se_3_-MW/Se-MT heterojunction devices. As the time interval from 10% to 90% of the maximum current of the device under illumination is defined as the rise time (t_r_), the time interval from 90% to 10% of the maximum current of the device after the light is removed is defined as the fall time (t_f_). The rise/fall time can well characterize the response speed of the optoelectronic device to the incident light. From the single-cycle I-t curve shown in Figure 5e, the rise/fall time of the Sb_2_Se_3_-MW/Se-MT/Ag-NW device is 8 and 10 ms at zero bias, respectively. From Figure 5f, it can be seen that the rise/fall time of the Sb_2_Se_3_-MW/Se-MT device is 0.32 and 0.12 s at zero bias, which is much larger. Therefore, the response of the Sb_2_Se_3_-MW/Se-MT/Ag-NW device is faster than that of the Sb_2_Se_3_-MW/Se-MT device; the Ag-NW-induced superior light absorption ability not only enhances the light current but also reduces the response time. The capacitance of the depletion region in the Sb_2_Se_3_-MW/Se-MT/Ag-NW device surpasses that of the Sb_2_Se_3_-MW/Se-MT device, resulting in a shorter photogenerated carrier transit time and a reduced device fall time.

To determine the optoelectronic capability of the devices, the key parameters of photoresponsivity (R_λ_) and the specific detectivity (D*) of the Sb_2_Se_3_-MW/Se-MT and the Sb_2_Se_3_-MW/Se-MT/Ag-NW device are evaluated. The photoresponsivity can be calculated by

(1) $$R_{\lambda }=\frac{I_{ph}}{P_{\lambda }S},$$

where I_ph_ is the photocurrent, P_λ_ is the optical power density of the incident light (368 nm of 0.52 mW cm^−2^, 800 nm of 0.30 mW cm^−2^, and 1000 nm of 0.28 mW cm^−2^), and S is the effective irradiated area of the device (7.6 × 10^−5^ cm^2^). The photocurrent can be determined by

(2) $$I_{ph}=I_{light}-I_{d},$$

where I_light_ and I_d_ denote the light and dark current, respectively. Specific detectivity, another important parameter of PDs, is calculated by [28]

(3) $$D^{*}=\frac{\sqrt{SB}}{NEP},$$

where B stands for the measurement bandwidth, and NEP is short for the noise equivalent power. Calculation details can be found in Figure S2 and Table S1.

**Figure 6:**
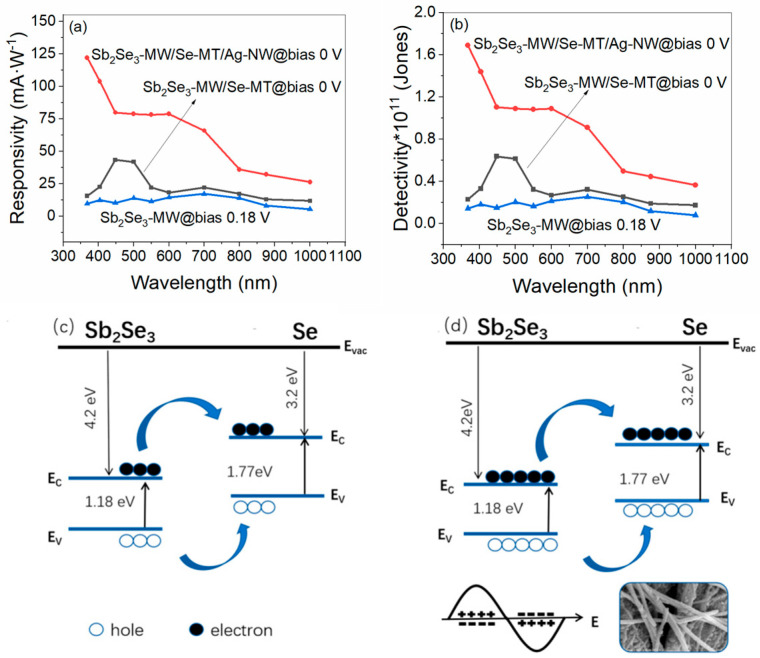
Responsivity curves (**a**) and specific detectivity curves (**b**) of the Sb_2_Se_3_-MW/Se-MT/Ag-NW and the Sb_2_Se_3_-MW/Se-MT devices at 0 V bias. The schematic diagrams of the energy band of the Sb_2_Se_3_-MW/Se-MT (**c**) and the Sb_2_Se_3_-MW/Se-MT/Ag-NW (**d**) devices.

As can be seen in Figure 6a, the calculated R_λ_ values for all three PDs vary with the wavelength. The responsivity value for the Sb_2_Se_3_-MW device at 0.18 V bias stays below 26 mA W^−1^ across the UV–vis range. Compared with the Sb_2_Se_3_-MW/Se-MT PD, the overall responsivity of the Sb_2_Se_3_-MW/Se-MT/Ag-NW device has a huge advantage. The maximal responsivity value for the Sb_2_Se_3_-MW/Se-MT/Ag-NW device reaches 122 mA W^−1^ at 368 nm and zero bias, which is 810% higher than that of the Sb_2_Se_3_-MW/Se-MT device, of which the value about 13.4 mA W^−1^. Here, it should be noticed that the maximum responsivity value (about 44 mA W^−1^) for the Sb_2_Se_3_-MW/Se-MT device is not at 368 nm but appears at 448 nm. The responsivity of the Sb_2_Se_3_-MW/Se-MT/Ag-NW device monotonically decreases while that of the Sb_2_Se_3_-MW/Se-MT device first increases and subsequently decreases as a function of wavelength. This can be explained by the decreasing penetration depth of photon light and the increasing recombination of the electron–hole carriers when wavelength becomes larger. Similarly, as can be seen in Figure 6b, the trend of the specific detectivity is basically consistent with the responsivity. The peak detectivity of the Sb_2_Se_3_-MW/Se-MT/Ag-NW PD at 368 nm and zero bias reaches 1.69 × 10^11^ Jones, which is 849% higher than that of the Sb_2_Se_3_-MW/Se-MT (1.78 × 10^10^ Jones), demonstrating its high capacity for tolerating the fixed noise when detecting optical signals in real applications. The above comparison results show that the LSPR effect stimulated by the addition of Ag-NW can well improve the responsivity and specific detectivity of Sb_2_Se_3_-MW/Se-MT heterojunction devices.

The proposed Sb_2_Se_3_-MW/Se-MT/Ag-NW PD exhibits the largest photocurrent, the highest on/off ratio, and the fastest response speed among all the three PDs. In terms of the key parameters like R_λ_ and D*, it also performs the best, showing a decisive edge over the competitors. To further reveal the LSPR of Ag-NW in the proposed PD, the working mechanism of Sb_2_Se_3_-MW/Se-MT and Sb_2_Se_3_-MW/Se-MT/Ag-NW devices is compared. In Figure 6c,d, the energy band diagrams of the two PDs are shown. The band gaps of the Sb_2_Se_3_-MW and the Se-MT are 1.18 eV [39] and 1.77 eV [24], respectively. The energy levels of the two in contact form a type II heterojunction. Under illumination conditions, the electrons in the valence band of the Se-MT are excited by the photons and jump to the conduction band, creating photogenerated electron–hole pairs. Due to the energy band difference between Se-MT and Sb_2_Se_3_-MW, the photogenerated electrons transfer from the conduction band of Se-MT to the conduction band of Sb_2_Se_3_-MW. At the same time, the holes in the valence band of Sb_2_Se_3_-MW also transfer to the valence band of Se-MT, completing the separation of the photogenerated electron–hole pairs, which are finally collected by the electrodes, thereby generating a larger photocurrent. After adding Ag-NW, the light absorption of Sb_2_Se_3_-MW/Se-MT was enhanced due to the LSPR effect. Comparing Figure 6d with Figure 6c, the Sb_2_Se_3_-MW/Se-MT/Ag-NW PD produces more electron–hole pairs under the influence of the LSPR effect; so, the photocurrent of the device is improved.

Table 1 lists the comparison of the main performance indicators of the PDs in this paper and others reported. Compared with other reported PDs in the table, the Sb_2_Se_3_-MW/Se-MT/Ag-NW device has great advantages in terms of the switch ratio, responsivity, and detection rate. At the same time, compared with the Sb_2_Se_3_-MW/Se-MT, the responsivity of the Sb_2_Se_3_-MW/Se-MT/Ag-NW device is improved overall, among which the peak responsivity at 368 nm under 0 V bias is 122 mA W^−1^, and the switch ratio reaches 618, which are 810% and 260% higher than Sb_2_Se_3_-MW/Se-MT, respectively. The LSPR effect stimulated by the addition of Ag-NW can greatly improve the photoelectric performance of the Sb_2_Se_3_-MW/Se-MT heterojunction device, which provides a new idea for further in-depth research on Sb_2_Se_3_-based PDs and utilization of the LSPR effect.

## 4. Conclusions

A Sb_2_Se_3_-MW/Se-MT/Ag-NW heterojunction was prepared by spray coating, and a photodetector was built. The photoelectric performance of the Sb_2_Se_3_-MW/Se-MT/Ag-NW heterojunction device was studied. By introducing Ag-NW and utilizing its LSPR effect, the light absorption capacity and photoelectric response of the device were significantly improved. Compared with Sb_2_Se_3_-MW/Se-MT, the overall responsivity of the Sb_2_Se_3_-MW/Se-MT/Ag-NW device was improved. The specific detectivity, peak responsivity, and on–off ratio at 368 nm under 0 V bias was 1.69 × 10^11^ Jones, 122 mA W^−1^, and 618, which are 849%, 810%, and 260% higher than those of Sb_2_Se_3_-MW/Se-MT, respectively. The experimental results show that the photocurrent and responsivity of the device at a specific wavelength are greatly improved, showing excellent photoelectric performance. In addition, the analysis of the energy band diagram reveals the working mechanism of the device, further confirming the key role of Ag-NW in improving the photoelectric performance. Compared with reported PDs, this device exhibits advantages in key performance indicators, showing broad application prospects in the field of photodetection and providing new ideas and methods for the design and preparation of high-performance PDs.

## Figures and Tables

**Table 1:** Comparison of the key performance of the Sb_2_Se_3_-based PDs proposed in this paper and others reported in recent years.

PD Device	Bias (V)	On/Off Ratio	Rise/Fall Time (ms)	R_λ_ (mA W ^−1^)	D* (Jones)	Ref.
Sb_2_Se_3_-MW/Se-MT	0	171	320/120	13.4	1.78 × 10^10^	This work
Sb_2_Se_3_-MW/Se-MT/Ag-NW	0	618	8/10	122	1.69 × 10^11^	This work
Sb_2_Se_3_-MW/ZnO	0	257	17/35	120	8.93 × 10^11^	[40]
Sb_2_Se_3_/Ga_2_O_3_	0	-	12/13	180	4.6 × 10^9^	[41]
Ni-Doped Sb_2_Se_3_	3	91	170/320	18.9	2.6 × 10^14^	[42]
MoS_2_/Sb_2_Se_3_/GaN	0	-	10/10	665	4.19 × 10^10^	[43]
Sb_2_Se_3_/Si	0	2000	1.7/2.9	25	1 × 10^10^	[44]
Sb_2_Se_3_/ZnO nanorod	0	415	-	137.17	1.33 × 10^9^	[12]
Selenized Sb_2_Se_3_	3	100	4.54/8.5	1130	4.62 × 10^11^	[45]
Sb_2_Se_3_/PEDOT	0	1804	23/60	2330	5.8 × 10^10^	[46]

## Data Availability

The original contributions presented in this study are included in the article/Supplementary Material. Further inquiries can be directed to the corresponding author(s).

## Supplementary Materials

The following supporting information can be downloaded at https://www.mdpi.com/article/10.3390/nano15241849/s1, Figure S1. I-t curves under different illumination conditions of the Sb_2_Se_3_-MW/Se-MT/Ag-NW (a) and the Sb_2_Se_3_-MW/Se-MT (b) at −4V bias. Figure S2. Noise power density (NPD) as a function of bias voltage of the Sb_2_Se_3_-MW/Se-MT/Ag-NW (a) and the Sb_2_Se_3_-MW/Se-MT (b) device. Table S1. Performance of the proposed devices at 0 and −4 V bias.
