# Supplementary material for: Ag Nanowires-Enhanced Sb2Se3 Microwires/Se Microtube Heterojunction for High Performance Self-Powered Broadband Photodetectors
Source: Nanomaterials (Basel). 2025 Dec 10;15(24):1849. doi: 10.3390/nano15241849 (PMC12736356; doi:10.3390/nano15241849)
Supplement: Supplementary file 1 [file nanomaterials-15-01849-s001.zip › nanomaterials-3996583-supplementary.pdf]

Supporting Information

Ag Nanowires Enhanced Sb_2Se_3 Microwires/Se Microtube Heterojunction for High Performance Self-Powered Broadband Photodetectors

*Shubin Zhang, Xiaonan Wang, Juntong Cui, Yanfeng Jiang, Pingping Yu**

School of Integrated Circuits, Jiangnan University, Wuxi 214122, China

The average I_{light} of the Sb_2Se_3-MW/Se-MT/Ag-NW device under –4 V bias and 368, 800, and 1000 nm light sources is about 61.8, 27.4, and 19.2 nA, respectively. Meanwhile, that of the Sb_2Se_3-MW/Se-MT device at –4 V bias is about 9.25, 3.51, and 2.33 nA. It is shown in Figure S1.

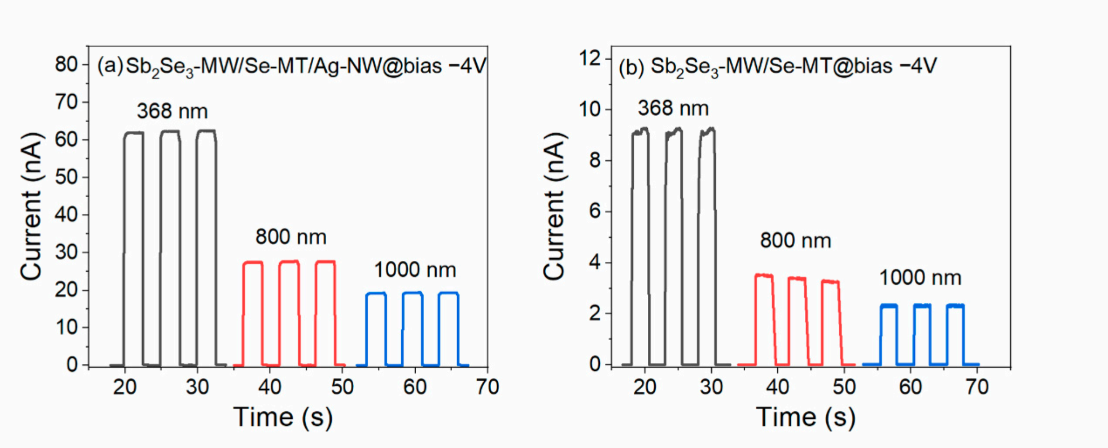

Figure S1. I-t curves under different illumination conditions of the Sb_2Se_3-MW/Se-MT/Ag-NW (a) and the Sb_2Se_3-MW/Se-MT (b) at −4V bias.

To calculate D*, noise power spectrum in a bandwidth of 1000 Hz is measured, as shown below in Figure S2.

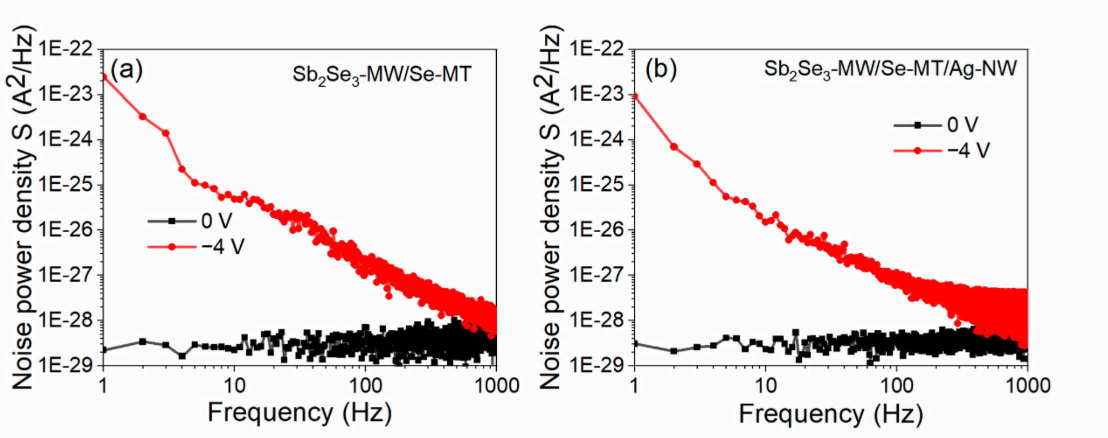

Figure S2. Noise power density (NPD) as a function of bias voltage of the Sb_2Se_3-MW/Se-MT/Ag-NW (a) and the Sb_2Se_3-MW/Se-MT (b) device.

Based on the data above, key performance parameters of the proposed devices at 0 and –4 V bias is listed in Table S1.

Table S1. Performance of the proposed devices at 0 and –4 V bias.

PD Device	Bias (V)	I_{Dark} (A)	I_{ph} (A)	NPD (A^2 Hz^{-1})	NEP (W $Hz^{-1/2}$)	R_λ (mA W^{-1})	D* (Jones)
Sb_2Se_3-MW/Se-MT/Ag-NW	0	7.78×10^{-12}	4.8×10^{-9}	3.91×10^{-29}	1.63×10^{-12}	122	1.69×10^{11}
Sb_2Se_3-MW/Se-MT	0	3.12×10^{-12}	5.3×10^{-10}	4.31×10^{-29}	1.55×10^{-11}	13.4	1.78×10^{10}
Sb_2Se_3-MW/Se-MT/Ag-NW	–4	6.33×10^{-10}	6.12×10^{-8}	1.16×10^{-25}	6.96×10^{-12}	1550	3.96×10^{10}
Sb_2Se_3-MW/Se-MT	–4	1.92×10^{-10}	9.06×10^{-9}	8.58×10^{-25}	1.28×10^{-10}	229	2.16×10^{9}
